# Evaluation of the bioMérieux VIDAS HIV Duo Quick and Anti-HCV assays for dried blood spot based serosurveillance

**DOI:** 10.1038/s41598-022-14041-z

**Published:** 2022-06-17

**Authors:** François Cholette, Braedy Farmer, Olga Balakireva, Daria Pavlova, Anna Lopatenko, Iryna Chukhalova, Svitlana Bargan, Sharmistha Mishra, Marissa Becker, Emma R. Lee, John Kim, Paul Sandstrom

**Affiliations:** 1 National HIV and Retrovirology Laboratories, National Microbiology Laboratory at the J.C. Wilt Infectious Diseases Research Centre, Public Health Agency of Canada, 745 Logan Avenue, Winnipeg, MB R3E 3L5 Canada; 2 Department of Medical Microbiology and Infectious Diseases, Max Rady College of Medicine, University of Manitoba, Winnipeg, Canada; 3 Institute for Economics and Forecasting, Ukrainian National Academy of Sciences, Kyiv, Ukraine; 4 Ukrainian Institute for Social Research After Oleksandr Yaremenko, Kyiv, Ukraine; 5 Dnipropetrovsk Oblast Medical Centre of Socially Significant Diseases, Dnipro, Ukraine; 6 MAP Centre for Urban Health Solutions, Li Ka Shing Knowledge Institute, St. Michael’s Hospital, Toronto, Canada; 7 Department of Medicine, University of Toronto, Toronto, Canada; 8 Institute of Medical Sciences, University of Toronto, Toronto, Canada; 9 Institute of Health Policy, Management and Evaluation, University of Toronto, Toronto, Canada; 10 Institute for Global Public Health, Department of Community Health Sciences, Rady Faculty of Health Sciences, University of Manitoba, Winnipeg, Canada

**Keywords:** Immunological techniques, Biological techniques, Biomarkers, Diagnostic markers

## Abstract

Serosurveillance is central to monitoring our progress towards HIV and HCV elimination targets proposed for 2030. However, serosurveillance systems are ineffective without reliable serological assays for the detection of HIV and HCV antibodies. Assays should also be compatible with dried blood spot (DBS) samples to facilitate biological sample collection. The VIDAS HIV Duo Quick and Anti-HCV assays are sold as reagents strips and processed by the automated VIDAS benchtop immunoanalyser. While both assays have shown excellent performance in serum and plasma, performance data in DBS samples is lacking. In our study, we evaluate the performance of the VIDAS HIV Duo Quick and Anti-HCV assays in DBS (*n* = 725) collected during a cross-sectional serosurvey (the *Transitions* study). The VIDAS HIV Duo quick had a sensitivity and specificity of 94.5% (95% CI 85.1%, 98.5%) and 95.7% (95% CI 93.9%, 97.0%) respectively. Likewise, the VIDAS Anti-HCV had a sensitivity and specificity of 95.6% (95% CI 91.6%, 97.8%) and 95.6% (95% CI 93.5%, 97.0%) respectively. These assays are unlikely to be helpful in low-prevalence settings due to sub-optimal performance, but their performance could likely be improved by optimizing DBS elution protocols which was, unfortunately, not possible during our study.

## Introduction

Human immunodeficiency virus (HIV) and hepatitis C virus (HCV) infections remain significant public health concerns globally^1,2^. Roughly 1.5 million people became newly infected with HIV and HCV in 2020 according to estimates from the Joint United Nations Programme on HIV/AIDS (UNAIDS)^3^ and the World Health Organization (WHO)^1^ respectively. The prevalence of HIV and HCV co-infection among key populations is also concerning, especially among people who inject drugs (PWID)^4,5^. Aided by highly active antiretroviral therapy (ART) and direct-acting antivirals (DAAs), ambitious targets for the elimination of HIV and HCV as public health threats have been set for 2030^1,6^. The UNAIDS 95-95-95 targets stipulate that 95% of people living with HIV (PLWH) should be aware of their HIV status, 95% of people who are aware of their status should be receiving treatment, and 95% of people on HIV treatment should be virally suppressed^6^. Along a similar vein, the WHO suggest we aim to treat 80% of HCV infections among those eligible, reduce the incidence of new HCV infections by 90%, and reduce liver-related mortality by 65%^1^. Progress towards these elimination targets can be monitored by behavioural and biological serosurveillance systems but, they necessitate accurate serological assays for the detection of HIV and HCV antibodies^7^. Serological assays should also be compatible with dried blood spot (DBS) samples to facilitate the implementation of surveillance by reducing structural and geographical barriers to biological specimen collection^8,9^.

The VIDAS HIV Duo Quick (bioMérieux, Marcy-l'Étoile, France) is a 4th generation enzyme-linked fluorescent assay (ELFA) capable of detecting the p24 antigen and antibodies to HIV^10^. The VIDAS Anti-HCV is a 3rd generation ELFA that makes use of synthetic core, NS3, NS4A, and NS4B antigens to detect antibodies to HCV^11^. Both assays are sold as ready-to-use strips containing all necessary reagents and protocols (i.e., washing, incubation, and detection) are automated by the VIDAS benchtop immunoanalyser thereby minimising operator error. Although the VIDAS system is considered low to medium throughput, it merits consideration due to its accessibility. The VIDAS system is simpler to operate compared to manual, 96-well plate based serological assays and cost significantly less ($50,000 CDN) than other commercial immunoanalysers like the Abbott Alinity or Bio-Rad BioPlex 2200 ($200,000 to $300,000 CDN). The VIDAS HIV Duo Quick and Anti-HCV have shown excellent performance in serum and plasma but, performance data in DBS are currently lacking especially in the context of serosurveillance^10–17^.

In our study, we evaluate the performance of the VIDAS HIV Duo Quick and Anti-HCV assay for the detection of HIV and HCV antibodies respectively in DBS for the purpose of serosurveillance. These assays were evaluated against a subset of DBS samples (*n* = 725) collected during a cross-sectional integrated biological and behavioural survey in the city of Dnipro, Ukraine. Sensitivity, specificity, positive predictive values, and negative predictive values were estimated using the Avioq HIV-1 Microelisa System or Ortho HCV v3.0 ELISA Test System as the reference test. Both reference tests are central to the Public Health Agency of Canada’s behavioural and biological surveillance system that monitors the prevalence of HIV and HCV among key populations in Canada using DBS samples^8^.

## Results

**Figure 1:**
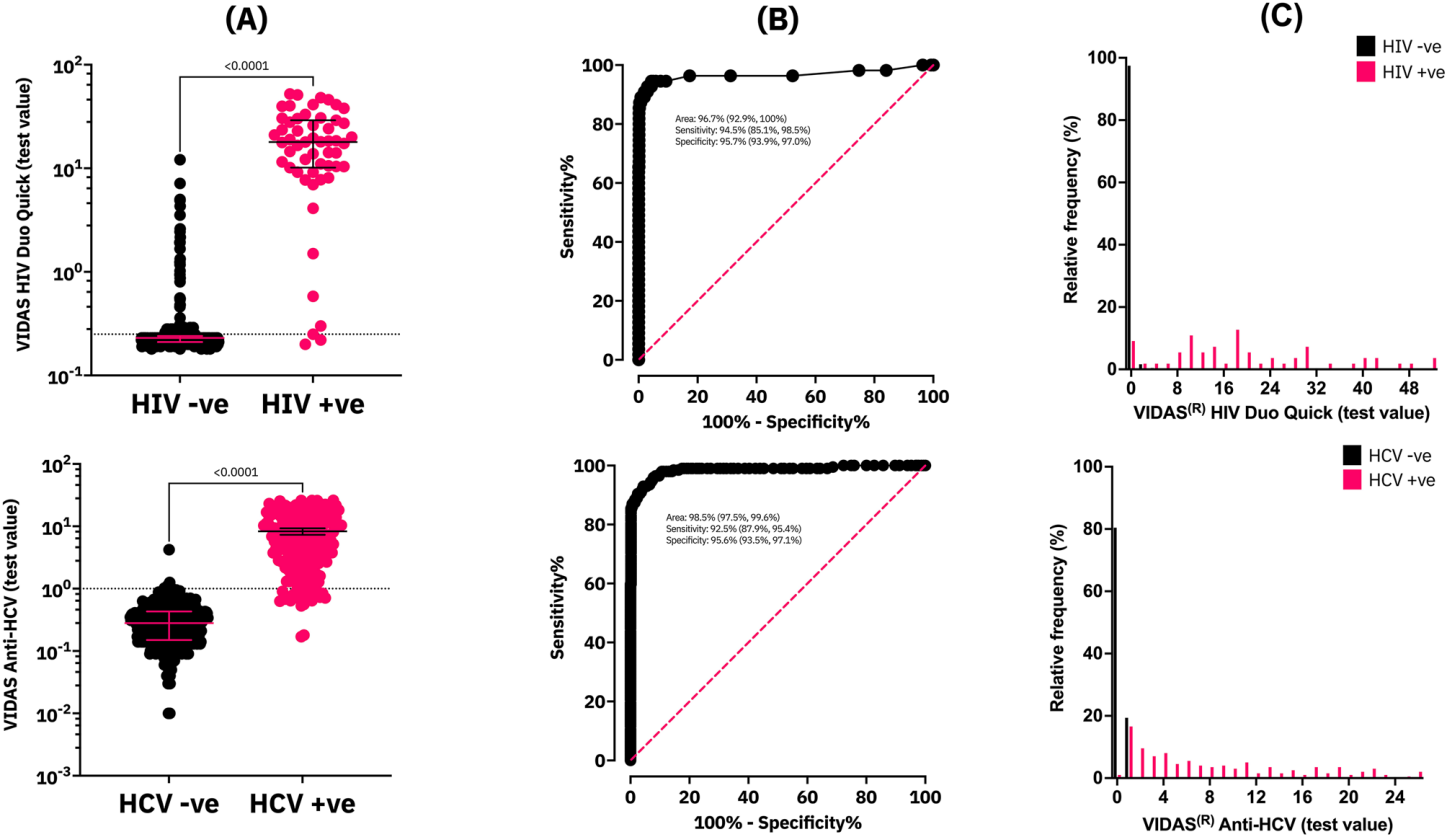
VIDAS HIV Duo Quick and Anti-HCV test values for DBS samples. (**A**) Median test values with interquartile ranges (IQR); median test value for HIV −ve DBS: 0.23 (IQR: 0.21, 0.24), median test value for HIV +ve DBS: 17.93 (IQR: 10.15, 29.12), median test value for HCV  −ve: 0.28 (IQR: 0.15, 0.43), median test value for HCV +ve: 5.99 (IQR: 2.23, 12.70); test value cutoffs recommend by the manufacturer are identified by the dotted lines (**B**) Receiver operating characteristic curves (**C**) Distribution of test values.

A random subset of DBS samples (*n* = 725) was chosen from the *Transitions* study to be tested retrospectively with the VIDAS HIV Duo Quick and Anti-HCV assays to assess their performance (Table 1). In this subset, the Avioq HIV-1 Microelisa System and Ortho v3.0 ELISA Test System were positive in 7.6% (55/725) and 27.4% (199/725) DBS samples respectively. The VIDAS HIV Duo Quick was positive in 96.4% (53/55) and negative in 82.7% (554/670) of the HIV positive and HIV negative DBS samples respectively corresponding to a sensitivity of 96.4% (95% CI 87.7%, 99.4%), specificity of 82.7% (95% CI 79.6%, 85.4%), PPV of 31.4% (95% CI 24.8%, 38.7%), and NPV of 99.6% (95% CI 98.7%, 99.9%). The VIDAS HIV Duo Quick was in moderate agreement (kappa = 0.405 [95% CI 0.326, 0.485]) with the Avioq HIV-1 Microelisa System based on kappa coefficients (Table 1). Figure 1 illustrates that the VIDAS HIV Duo Quick can adequately discriminate between HIV positive and HIV negative DBS. Furthermore, receiver operating characteristic (ROC) curve analysis suggested that performance could be improved by adjusting the test value cutoff provided by the manufacturer (Fig. 1). Adjusting the test value significantly improved specificity (95.7% [95% CI 93.9%, 97.0%] versus 82.7% [95% CI 79.6%, 85.4%]) and agreement with the Avioq HIV-1 Microelisa System (0.741 [95% CI 0.656, 0.826] versus 0.405 [95% CI 0.326, 0.485]).

**Table 1:** Performance statistics for the detection of HIV and HCV antibodies in DBS samples using the VIDAS HIV Duo Quick and Anti-HCV.

	VIDAS HIV Duo Quick		VIDAS Anti-HCV	
Cutoff (test value)	0.25^a^	0.30^b^	1.00^a^	0.80^b^
Sensitivity (*n*/N)% (95% CI)	53/55 96.4 (87.7, 99.4)	52/55 94.5 (85.1, 98.5)	175/199 87.9 (82.7, 91.8)	185/199 93.0 (88.5, 95.8)
Specificity (*n*/N) % (95% CI)	554/670 82.7 (79.6, 85.4)	641/670 95.7 (93.9, 97.0)	518/526 98.5 (97.0, 99.2)	503/526 95.6 (93.5, 97.1)
PPV (*n*/N) % (95% CI)	116/670 31.4 (24.8, 38.7)	29/670 64.2 (53.3, 73.8)	8/526 95.6 (91.6, 0.97.8)	23/526 88.9 (84.0, 92.5)
NPV (*n*/N) % (95% CI)	2/55 99.6 (98.7, 99.9)	3/55 99.5 (98.6, 99.9)	24/199 95.6 (93.5, 97.0)	14/199 97.3 (95.5, 98.4)
Kappa (95% CI)	0.405 (0.326, 0.485)	0.741 (0.656, 0.826)	0.886 (0.848, 0.925)	0.874 (0.834, 0.913)

^a^Test value recommended by the manufacturer.

^b^Test value cutoff determined by ROC curve analysis.

The VIDAS Anti-HCV was positive in 87.9% (175/199) and negative in 98.5% (518/526) of the HCV positive and HCV negative DBS samples respectively corresponding to a sensitivity of 95.6% (95% CI 91.6%, 97.8%), specificity of 95.6% (95% CI 93.5%, 97.0%), PPV of 95.6% (95% CI 91.6%, 97.8%), and NPV of 95.6% (93.5%, 97.0%). The VIDAS Anti-HCV was in almost perfect agreement with the Ortho HCV v3.0 ELISA Test System based on kappa coefficients (kappa = 0.886 [95% CI 0.848, 0.925]). The VIDAS Anti-HCV could also adequately discriminate between HCV positive and HCV negative DBS (Fig. 1) however, adjusting the test value cutoff did not significantly impact performance (Table 1).

## Discussion

Serosurveillance will play a central role locally and internationally in monitoring progress made towards HIV and HCV elimination targets set for 2030^1,6^. To be effective, serosurveillance systems require accurate serological assays for the detection of HIV and HCV antibodies^7^. While serum and plasma are typically the preferred biological specimen for serosurveillance, collection is not always convenient or possible due to a lack of infrastructure^9,18^, shortages of trained personnel (i.e., phlebotomists)^19,20^, and barriers to accessing collection sites^21^. Serological assays should also perform satisfactorily with DBS samples to facilitate biological sample collection. This may lead to more representative sampling and accurate estimates of disease prevalence.

The VIDAS HIV Duo quick had a sensitivity and specificity of 94.5% (95% CI 85.1%, 98.5%) and 95.7% (95% CI 93.9%, 97.0%) respectively. This is considerably lower compared to performance reported by others using DBS. In a study conducted among PLWH on ART in Kinshasa, Democratic Republic of Congo Barquín et al.^12^ reported a sensitivity of 100% (95% CI 98.6%, 100%) and specificity of 100% (95% CI 96.0%, 100%) for the VIDAS HIV Duo Quick. Differences in performance between our study and Barquín et al*.* could be explained by procedural differences. In the Barquín et al*.* study, blood was collected by venipuncture and spotted onto 903 Protein Saver Cards with a micropipette. The DBS samples in our study were directly spotted using finger-pokes. Serological assays in general may perform better with DBS samples prepared from venous blood versus finger-pokes^22^. Furthermore, Barquín et al*.* eluted entire DBS spots (70 µL) in phosphate buffered saline (PBS) while we eluted a single 6 mm DBS punch (~ 20 µL) in PBS containing 0.5% BSA and 0.05% Tween 20. Sample input (e.g., quantity of punches) and choice of elution buffer significantly impact performance^23^. Furthermore, the Avioq HIV-1 Microelisa System could have missed acute HIV infections, thereby impacting our sensitivity and specificity calculations. The VIDAS HIV Duo Quick allows for the simultaneous detection of p24 antigen and anti-HIV antibodies while the Avioq HIV-1 Microelisa System only detects anti-HIV antibodies. We were unable to confirm the presence of acute HIV infections at the time of this study due to a lack of sample availability for nucleic acid testing. However, we are confident that acute HIV infections had a minimal impact on our performance calculations. It has been estimated that the prevalence of acute HIV infections ranges from 1% to 8.3% depending on the study population^24^. Therefore, we expect the proportion of acute infections to be relatively low in our study population. Although our findings suggest that the VIDAS Duo Quick may have limited use in low prevalence settings due to a PPV of 64.2% (95% CI 53.3%, 73.8%), optimizing our elution protocol for DBS samples and diagnosing acute HIV infections is likely warranted to achieve a better estimate of the assay’s performance. This was not possible during our study due to limited sample volume but, future work will consist of improving our elution protocol using DBS samples contrived^9^ from highly characterized reference serum/plasma samples^25^.

In contrast, the VIDAS Anti-HCV performed better in our laboratory compared to a study by Carrasco et al*.*^13^ In this study, DBS samples were collected from adult patients attending hospitals in Kinshasa, Democratic Republic of Congo as part of the Observational Kinshasha AIDS Prevention Initiative (OKAPI) project. The authors reported a sensitivity and specificity of 61.5% (95% CI 31.6%, 86.1%) and 89.4% (95% CI 81.3%, 94.8%) respectively for the VIDAS Anti-HCV. Furthermore, their findings would suggest that the VIDAS Anti-HCV would be of limited use for serosurveillance due to a PPV and NPV of 44.4% (95% CI 21.5%, 69.2%) and 94.4% (95% CI 87.4%, 98.1%) respectively. It would be important to note however, that Carrasco et al*.* lacked a reference test and relied on two positive/indeterminate results among the three index tests under evaluation to establish “true positives”. Even though our findings are more encouraging, improving our DBS elution protocol will likely be necessary to improve performance and make use of the VIDAS Anti-HCV in low-prevalence settings.

In the present study, we evaluated the performance of the VIDAS HIV Duo Quick and Anti-HCV assay for the detection of HIV and HCV antibodies respectively in DBS samples for the purpose of serosurveillance. These assays are unlikely to be helpful in low-prevalence settings due to less-than-ideal PPV and NPV. However, it is possible that optimizing DBS elution protocols (e.g., quantity of punches, choice of elution buffer) will improve performance but was not possible during our study. Improving our elution protocols will be investigated in future work with the help of highly characterized reference material.

## Methods

### Study participants and sample collection

The *Transitions* study is a cross-sectional integrated biological and behavioural survey conceived to measure the prevalence and patterns of HIV risk and vulnerabilities^26–28^. From March to December 2015, field teams in the city of Dnipro, Ukraine administered written informed consent, conducted interviews, and oversaw multiplex rapid testing for HIV, syphilis, and hepatitis B and C (Profitest Combo, New Vision Diagnostics, Haicang Xiamen, China) among study participants. DBS samples were also collected from consenting participants on 903 Protein Saver Cards (Cytiva Whatman, Marlborough, MA) using Microtainer high flow contact activated lancets (BD, Franklin Lakes, NJ) for HIV and HCV confirmatory serological testing. Confirmatory testing was performed by the National HIV and Retrovirology Laboratories of the Public Health Agency of Canada (Winnipeg, Canada) using the Avioq HIV-1 Microelisa System (Avioq Inc., Durham, NC) and Ortho HCV v3.0 ELISA Test System (Ortho-Clinical Diagnostics Inc., Raritan, NJ). We enrolled 1,818 participants and all participants consented to providing a DBS sample. A subset of DBS samples (*n* = 725 [39.9%]) were randomly chosen to evaluate the performance of the bioMérieux VIDAS HIV Duo Quick and Anti-HCV assays with DBS samples.

### Avioq HIV-1 Microelisa System

The Avioq HIV-1 Microelisa System (Avioq Inc., Research Triangle Park, NC) was used as the reference test for the qualitative detection of antibodies to HIV. DBS samples were punched (1 X 6 mm) using a pneumatic Dried Blood Spot Punch System (Analytical Sales and Services Inc., Flanders, NJ) into 2 mL 96-well polypropylene plates (ThermoFisher Scientific, Ottawa, Canada) and processed according to instructions provided by the manufacturer. Cutoff values (COV) were calculated as follows: COV = NCX + 0.270 where NCX represents the mean of the Negative Calibrator (Avioq Inc.) values. A result < COV and ≥ COV was interpreted as negative and positive respectively.

### Ortho HCV v3.0 ELISA Test System

The Ortho HCV v3.0 ELISA Test System (Ortho-Clinical Diagnostics Inc., Raritan, NJ) was used as the reference test for the qualitative detection of antibodies to HCV. DBS specimens were punched (1 X 6 mm) using a pneumatic Dried Blood Spot Punch System (Analytical Sales and Services Inc.) into 2 mL 96-well polypropylene plates (ThermoFisher Scientific). DBS punches were eluted in 200 µL of Specimen Diluent (Ortho-Clinical Diagnostics Inc.) overnight at 4 °C without agitation. Afterwards, plates were gently agitated (400 rpm) at room temperature for 30 min and 150 µL of DBS eluate was transferred into HCV Encoded Antigen Coated Microwell Plates (Ortho-Clinical Diagnostics Inc.) containing 50 µL of Specimen Diluent (Ortho-Clinical Diagnostics Inc.) and processed according to instructions provided by the manufacturer. COV were calculated as follows: COV = NCX + 0.600 where NCX represents the mean of the Negative Control (Ortho-Clinical Diagnostics Inc.) values. The upper and lower limit of the grey zone (UGZ and LGZ respectively) were calculated as follows: UGZ = COV X 1.5 and LGZ = COV X 0.9. A result < LGZ, ≥ LGZ to < UGZ, and ≥ UGZ was interpreted as negative, indeterminate, and positive respectively.

### VIDAS HIV Duo Quick

The VIDAS HIV Duo Quick (bioMérieux) was used as the index test for the qualitative detection of antibodies to HIV. DBS specimens were punched (1 X 6 mm) using a pneumatic Dried Blood Spot Punch System (Analytical Sales and Services Inc.) into 2 mL 96-well polypropylene plates (ThermoFisher Scientific). DBS punches were eluted in 200 µL of DPBS pH 7.4 containing 0.5% BSA and 0.05% Tween 20 at room temperature for 1 h with agitation (1000 rpm). The elution buffer is based on some of our previous work^9^. The punching and elution protocol was carried out according to the Avioq HIV-1 Microelisa System package insert and took into consideration the minimal input volume for the VIDAS HIV Duo Quick test. Afterwards, all remaining DBS eluate was transferred into HIV6 Strips (bioMérieux) and processed by an automated VIDAS benchtop immunoanalyzer (bioMérieux). A test value < 0.25 and ≥ 0.25 was interpreted as negative and positive respectively unless stated otherwise. Test values (TV) were calculated as follows: TV = relative fluorescent value/background fluorescence value.

### VIDAS Anti-HCV

The VIDAS Anti-HCV (bioMérieux) was used as the index test for the qualitative detection of antibodies to HCV. DBS specimens were punched (1 X 6 mm) using a pneumatic Dried Blood Spot Punch System (Analytical Sales and Services Inc.) Into 2 mL 96-well polypropylene plates (ThermoFisher Scientific). DBS punches were eluted in 100 µL of DPBS pH 7.4 containing 0.5% BSA and 0.05% Tween 20 at room temperature for 1 h with agitation (1000 rpm) as indicated above. Afterwards, all remaining DBS eluate was transferred into HCV Strips (bioMérieux) and processed by an automated VIDAS benchtop immunoanalyzer (bioMérieux). A test value < 1.0 and ≥ 1.0 was interpreted as negative and positive respectively unless stated otherwise. Test values (TV) were calculated as follows: TV = relative fluorescent value / background fluorescence value.

### Statistical analysis

Continuous data were summarized using the median and interquartile range (IQR). Categorical data were presented using exact numbers and proportions. Sensitivity, specificity, negative predictive values (NPV), and positive predictive values (PPV) were estimated using the Avioq HIV-1 Microelisa System or Ortho HCV v3.0 ELISA Test System as the reference test.

The strength of agreement between the index test (VIDAS HIV Duo Quick or Anti-HCV) and the corresponding reference test was quantified with kappa coefficients (https://www.graphpad.com/quickcalcs/kappa1/?K=2). Kappa coefficients were interpreted as follows: < 0 = no agreement, 0–0.20 = slight agreement, 0.21–0.40 = fair agreement, 0.41–0.60 = moderate agreement, 0.61–0.80 = substantial agreement, and 0.81–1.00 = almost perfect agreement^29^.

Cutoff values for the VIDAS HIV Duo Quick and Anti-HCV assays were determined by using a combination of receiver operating characteristic (ROC) curves and Youden's J statistic^30^. Youden's J statistic was computed as follows: J = sensitivity + specificity − 1. Prism v9.0.0 (GraphPad Software, San Diego, CA) was used for all data analysis and visualisation.

### Ethics

The Transition study was reviewed and approved by the Human Research Ethics Board at the University of Manitoba in Canada (HS16557 [H2013:295]), the Ethical Review Committee Board at the Sociological Association of Ukraine, Committee on Medical Ethics of the L. Gromashevsky Institute of Epidemiology and Infectious Diseases at the National Academy of Medical Sciences of Ukraine. All experiments were carried out in accordance with relevant guidelines and regulations. All participants provided written informed consent. Participants under 16 years of age were considered mature minors and provided consent to take part in the study without consent from their parent or guardian. Written informed consent from the participant’s legal guardian/next of kin was not required to participate in this study in accordance with the local legislation and the Human Research Ethics Board at the University of Manitoba in Canada and the Ethical Review Committee Board at the Sociological Association of Ukraine, Committee on Medical Ethics of the L. Gromashevsky Institute of Epidemiology and Infectious Diseases at the National Academy of Medical Sciences of Ukraine.

## Supplementary Information

Supplementary Tables.

## Data Availability

All data generated or analysed during this study are included in this published article (and its Supplementary Information files).
