# Supplementary material for: Evaluation of the bioMérieux VIDAS HIV Duo Quick and Anti-HCV assays for dried blood spot based serosurveillance
Source: Sci Rep. 2022 Jun 17;12:10171. doi: 10.1038/s41598-022-14041-z (PMC9205869; doi:10.1038/s41598-022-14041-z)
Supplement: Supplementary file 1 — Supplementary Tables. [file 41598_2022_14041_MOESM1_ESM.docx]

**Table 1. Raw data generated from dried blood samples.**

| **Sample no.** | **Avioq® HIV-1 Microelisa System**  **(Optical density)** | **Ortho® HCV v3.0 ELISA Test System**  **(Optical density)** | **VIDAS® HIV Duo Quick**  **(Test value)** | **VIDAS® Anti-HCV**  **(Test value)** |
| --- | --- | --- | --- | --- |
| TS15-DBS-001 | 0.1316 | 0.2586 | 0.23 | 0.25 |
| TS15-DBS-002 | 0.1198 | 0.4702 | 0.22 | 0.28 |
| TS15-DBS-003 | 0.1406 | 0.2212 | 0.23 | 0.39 |
| TS15-DBS-004 | 0.1211 | 0.237 | 0.23 | 0.32 |
| TS15-DBS-005 | 0.1167 | 0.1775 | 0.23 | 0.34 |
| TS15-DBS-006 | 0.1309 | 0.2078 | 0.2 | 0.36 |
| TS15-DBS-007 | 0.141 | 0.2122 | 0.21 | 0.32 |
| TS15-DBS-008 | 0.1501 | 0.2031 | 0.19 | 0.35 |
| TS15-DBS-009 | 0.1336 | 0.1118 | 0.22 | 0.26 |
| TS15-DBS-010 | 0.1088 | 0.1959 | 0.21 | 0.37 |
| TS15-DBS-011 | 0.1701 | 0.1405 | 0.23 | 0.08 |
| TS15-DBS-012 | 0.109 | 0.1321 | 0.24 | 0.13 |
| TS15-DBS-013 | 0.1255 | 0.1083 | 0.23 | 0.09 |
| TS15-DBS-014 | 0.1322 | 0.2063 | 0.23 | 0.26 |
| TS15-DBS-015 | 0.1538 | 0.1806 | 0.23 | 0.25 |
| TS15-DBS-016 | 0.14 | 0.1213 | 0.21 | 0.13 |
| TS15-DBS-017 | 0.1403 | 0.1268 | 0.23 | 0.21 |
| TS15-DBS-018 | 0.0962 | 0.1039 | 0.23 | 0.11 |
| TS15-DBS-019 | 0.1369 | 0.1238 | 0.26 | 0.15 |
| TS15-DBS-020 | 0.1068 | 0.1177 | 0.27 | 0.1 |
| TS15-DBS-021 | 0.1336 | 0.1192 | 0.26 | 0.09 |
| TS15-DBS-022 | 0.1148 | 0.1484 | 0.24 | 0.06 |
| TS15-DBS-023 | 0.1362 | 0.2123 | 0.21 | 0.33 |
| TS15-DBS-024 | 0.1268 | 0.1694 | 0.23 | 0.28 |
| TS15-DBS-025 | 0.1149 | 0.1612 | 0.22 | 0.32 |
| TS15-DBS-026 | 0.1207 | 0.3126 | 0.26 | 0.47 |
| TS15-DBS-027 | 0.1076 | 0.2301 | 0.27 | 0.06 |
| TS15-DBS-028 | 0.1107 | 0.3157 | 0.24 | 0.33 |
| TS15-DBS-029 | 0.1075 | 0.1609 | 0.23 | 0.08 |
| TS15-DBS-030 | 0.1166 | 0.2305 | 0.2 | 0.53 |
| TS15-DBS-031 | 0.1286 | 0.1958 | 0.26 | 0.34 |
| TS15-DBS-032 | 0.1145 | 0.2464 | 0.2 | 0.16 |
| TS15-DBS-033 | 0.1009 | 0.2714 | 0.18 | 0.09 |
| TS15-DBS-034 | 0.1056 | 0.2487 | 0.21 | 0.28 |
| TS15-DBS-035 | 0.1057 | 0.2495 | 0.22 | 0.48 |
| TS15-DBS-036 | 0.1474 | 0.1745 | 0.2 | 0.3 |
| TS15-DBS-037 | 0.1706 | 0.2008 | 0.23 | 0.14 |
| TS15-DBS-038 | 0.1418 | 0.2412 | 0.22 | 0.49 |
| TS15-DBS-039 | 0.1334 | 0.2374 | 0.21 | 0.11 |
| TS15-DBS-040 | 0.1114 | 0.1299 | 0.2 | 0.12 |
| TS15-DBS-041 | 0.1229 | 0.1648 | 0.22 | 0.18 |
| TS15-DBS-042 | 0.1062 | 0.172 | 0.22 | 0.11 |
| TS15-DBS-043 | 0.116 | 0.1479 | 0.24 | 0.11 |
| TS15-DBS-044 | 0.1112 | 0.1719 | 0.23 | 0.14 |
| TS15-DBS-045 | 0.0987 | 0.1967 | 0.24 | 0.12 |
| TS15-DBS-046 | 0.1152 | 0.1978 | 0.23 | 0.19 |
| TS15-DBS-047 | 0.1313 | 0.1676 | 0.21 | 0.14 |
| TS15-DBS-048 | 0.1049 | 0.3121 | 0.24 | 0.17 |
| TS15-DBS-049 | 0.1214 | 0.3369 | 0.23 | 0.11 |
| TS15-DBS-050 | 0.1113 | 0.2774 | 0.24 | 0 |
| TS15-DBS-051 | 0.117 | 0.1337 | 0.23 | 0.03 |
| TS15-DBS-052 | 0.1224 | 0.2394 | 0.29 | 0.19 |
| TS15-DBS-053 | 0.1053 | 0.3198 | 0.25 | 0.21 |
| TS15-DBS-054 | 0.1236 | 0.2492 | 0.23 | 0.14 |
| TS15-DBS-055 | 0.1335 | 0.4117 | 0.23 | 0.16 |
| TS15-DBS-056 | 0.1164 | 0.3053 | 0.24 | 0.29 |
| TS15-DBS-057 | 0.1337 | 0.171 | 0.2 | 0.11 |
| TS15-DBS-058 | 0.1107 | 0.1956 | 0.2 | 0.16 |
| TS15-DBS-059 | 0.131 | 0.1877 | 0.24 | 0.16 |
| TS15-DBS-060 | 0.1573 | 0.1701 | 0.22 | 0.11 |
| TS15-DBS-061 | 0.1436 | 0.2448 | 0.23 | 0.17 |
| TS15-DBS-062 | 0.1191 | 0.0977 | 0.22 | 0.03 |
| TS15-DBS-063 | 0.1247 | 0.1607 | 0.2 | 0.16 |
| TS15-DBS-064 | 0.1219 | 0.2418 | 0.23 | 0.13 |
| TS15-DBS-065 | 0.1342 | 0.1706 | 0.23 | 0.18 |
| TS15-DBS-066 | 0.1232 | 0.1722 | 0.24 | 0.1 |
| TS15-DBS-067 | 0.1422 | 0.1737 | 0.19 | 0.78 |
| TS15-DBS-068 | 0.125 | 0.24 | 0.22 | 1.04 |
| TS15-DBS-069 | 0.1483 | 0.1812 | 0.21 | 0.85 |
| TS15-DBS-070 | 0.1449 | 0.1498 | 0.2 | 0.83 |
| TS15-DBS-071 | 0.1287 | 0.2679 | 0.23 | 1.08 |
| TS15-DBS-072 | 0.1433 | 0.2572 | 0.23 | 0.85 |
| TS15-DBS-073 | 0.1559 | 0.2446 | 0.21 | 0.69 |
| TS15-DBS-074 | 0.1295 | 0.2222 | 0.22 | 0.81 |
| TS15-DBS-075 | 0.1203 | 0.2778 | 0.28 | 1.1 |
| TS15-DBS-076 | 0.1273 | 0.3094 | 0.22 | 0.66 |
| TS15-DBS-077 | 0.1453 | 0.2084 | 0.22 | 0.56 |
| TS15-DBS-078 | 0.1736 | 0.2177 | 0.19 | 0.64 |
| TS15-DBS-079 | 0.1357 | 0.3001 | 0.24 | 0.59 |
| TS15-DBS-080 | 0.1474 | 0.1782 | 0.24 | 0.36 |
| TS15-DBS-081 | 0.1048 | 0.158 | 0.23 | 0.55 |
| TS15-DBS-082 | 0.1088 | 0.2024 | 0.24 | 0.44 |
| TS15-DBS-083 | 0.1161 | 0.2822 | 0.23 | 0.55 |
| TS15-DBS-084 | 0.1143 | 0.2654 | 0.2 | 0.56 |
| TS15-DBS-085 | 0.1318 | 0.1201 | 0.48 | 0.21 |
| TS15-DBS-086 | 0.1029 | 0.0933 | 0.22 | 0.12 |
| TS15-DBS-087 | 0.14 | 0.7718 | 0.23 | 0.62 |
| TS15-DBS-088 | 0.1273 | 0.0967 | 0.21 | 0.08 |
| TS15-DBS-089 | 0.1156 | 0.1004 | 0.25 | 0.07 |
| TS15-DBS-090 | 0.1298 | 0.1787 | 0.2 | 0.07 |
| TS15-DBS-091 | 0.099 | 0.2105 | 0.23 | 0.12 |
| TS15-DBS-092 | 0.1229 | 0.2572 | 0.19 | 0.11 |
| TS15-DBS-093 | 0.1047 | 0.1965 | 0.25 | 0.07 |
| TS15-DBS-094 | 0.1046 | 0.0998 | 0.2 | 0.1 |
| TS15-DBS-095 | 0.0945 | 0.1059 | 0.25 | 0.11 |
| TS15-DBS-096 | 0.1156 | 0.1019 | 0.22 | 0.03 |
| TS15-DBS-097 | 0.1184 | 0.1357 | 0.22 | 0.64 |
| TS15-DBS-098 | 0.1339 | 0.1573 | 0.23 | 0.77 |
| TS15-DBS-099 | 0.1171 | 0.2161 | 0.23 | 0.75 |
| TS15-DBS-100 | 0.11 | 0.2297 | 0.2 | 0.37 |
| TS15-DBS-101 | 0.1228 | 0.2065 | 0.22 | 0.53 |
| TS15-DBS-102 | 0.1218 | 0.1729 | 0.22 | 0.41 |
| TS15-DBS-103 | 0.1207 | 0.1367 | 0.24 | 1.2 |
| TS15-DBS-104 | 0.1324 | 0.1517 | 0.2 | 0.67 |
| TS15-DBS-105 | 0.1068 | 0.1537 | 0.21 | 0.61 |
| TS15-DBS-106 | 0.1376 | 0.1832 | 0.2 | 0.47 |
| TS15-DBS-107 | 0.1204 | 0.1741 | 0.2 | 0.59 |
| TS15-DBS-108 | 0.1266 | 0.1852 | 0.22 | 0.5 |
| TS15-DBS-109 | 0.1303 | 0.1639 | 0.21 | 0.36 |
| TS15-DBS-110 | 0.1387 | 0.1729 | 0.22 | 0.48 |
| TS15-DBS-111 | 0.1366 | 0.135 | 0.21 | 0.58 |
| TS15-DBS-112 | 0.1242 | 0.148 | 0.2 | 0.37 |
| TS15-DBS-113 | 0.1251 | 0.1211 | 0.24 | 0.16 |
| TS15-DBS-114 | 0.1286 | 0.1929 | 0.21 | 0.25 |
| TS15-DBS-115 | 0.1181 | 0.2032 | 0.26 | 0.26 |
| TS15-DBS-116 | 0.1251 | 0.176 | 0.21 | 0.38 |
| TS15-DBS-117 | 0.1214 | 0.166 | 0.24 | 0.48 |
| TS15-DBS-118 | 0.2192 | 0.1494 | 0.2 | 0.55 |
| TS15-DBS-119 | 0.1284 | 0.1499 | 0.21 | 0.32 |
| TS15-DBS-120 | 0.1427 | 0.139 | 0.22 | 0.37 |
| TS15-DBS-121 | 0.189 | 0.2146 | 0.21 | 0.58 |
| TS15-DBS-122 | 0.1481 | 0.162 | 0.21 | 0.41 |
| TS15-DBS-123 | 0.1482 | 0.2204 | 0.24 | 0.43 |
| TS15-DBS-124 | 0.1776 | 0.1228 | 0.22 | 0.76 |
| TS15-DBS-125 | 0.1419 | 0.174 | 0.25 | 0.41 |
| TS15-DBS-126 | 0.1055 | 0.1283 | 0.22 | 0.3 |
| TS15-DBS-127 | 0.1358 | 0.1903 | 0.22 | 0.92 |
| TS15-DBS-128 | 0.126 | 0.1448 | 0.24 | 0.68 |
| TS15-DBS-129 | 0.122 | 0.1204 | 0.22 | 0.79 |
| TS15-DBS-130 | 0.1571 | 0.1099 | 0.24 | 0.53 |
| TS15-DBS-131 | 0.1076 | 0.1022 | 0.27 | 0.28 |
| TS15-DBS-132 | 0.1162 | 0.1508 | 0.21 | 0.64 |
| TS15-DBS-133 | 0.131 | 0.1195 | 0.22 | 0.26 |
| TS15-DBS-134 | 0.1133 | 0.1297 | 0.22 | 0.44 |
| TS15-DBS-135 | 0.1266 | 0.1228 | 0.25 | 0.35 |
| TS15-DBS-136 | 0.1081 | 0.1046 | 0.22 | 0.28 |
| TS15-DBS-137 | 0.1171 | 0.1356 | 0.23 | 0.28 |
| TS15-DBS-138 | 0.1285 | 0.1288 | 0.22 | 0.22 |
| TS15-DBS-139 | 0.1192 | 0.2154 | 0.22 | 0.42 |
| TS15-DBS-140 | 0.1861 | 0.242 | 0.23 | 0.65 |
| TS15-DBS-141 | 0.1183 | 0.1545 | 0.24 | 0.37 |
| TS15-DBS-142 | 0.1426 | 0.2239 | 0.2 | 0.42 |
| TS15-DBS-143 | 0.149 | 0.2148 | 0.22 | 0.42 |
| TS15-DBS-144 | 0.1129 | 0.2173 | 0.22 | 0.44 |
| TS15-DBS-145 | 0.1311 | 0.1759 | 0.22 | 0.43 |
| TS15-DBS-146 | 0.1493 | 0.2027 | 0.2 | 0.52 |
| TS15-DBS-147 | 0.152 | 0.1666 | 0.22 | 0.47 |
| TS15-DBS-148 | 0.1816 | 0.1822 | 0.2 | 0.38 |
| TS15-DBS-149 | 0.1207 | 0.2118 | 0.23 | 0.41 |
| TS15-DBS-150 | 0.1325 | 0.182 | 0.22 | 0.38 |
| TS15-DBS-151 | 0.1224 | 0.1728 | 0.23 | 0.35 |
| TS15-DBS-152 | 0.122 | 0.1585 | 0.23 | 0.51 |
| TS15-DBS-153 | 0.15 | 0.203 | 0.25 | 0.53 |
| TS15-DBS-154 | 0.1151 | 0.2119 | 0.23 | 0.55 |
| TS15-DBS-155 | 0.117 | 0.2296 | 0.22 | 0.52 |
| TS15-DBS-156 | 0.1376 | 0.2934 | 0.24 | 0.57 |
| TS15-DBS-157 | 0.1202 | 0.1925 | 0.19 | 0.76 |
| TS15-DBS-158 | 0.1356 | 0.1782 | 0.22 | 0.67 |
| TS15-DBS-159 | 0.1722 | 0.1661 | 0.22 | 0.4 |
| TS15-DBS-160 | 0.1238 | 0.1874 | 0.21 | 0.43 |
| TS15-DBS-161 | 0.1231 | 0.1452 | 0.19 | 0.33 |
| TS15-DBS-162 | 0.1598 | 0.1855 | 0.23 | 0.46 |
| TS15-DBS-163 | 0.121 | 0.225 | 0.24 | 0.54 |
| TS15-DBS-164 | 0.1391 | 0.1976 | 0.24 | 0.42 |
| TS15-DBS-165 | 0.1335 | 0.2017 | 0.2 | 0.33 |
| TS15-DBS-166 | 0.1127 | 0.173 | 0.22 | 0.43 |
| TS15-DBS-167 | 0.1494 | 0.151 | 0.22 | 0.29 |
| TS15-DBS-168 | 0.1221 | 0.1662 | 0.25 | 0.55 |
| TS15-DBS-169 | 0.1119 | 0.2025 | 0.2 | 0.81 |
| TS15-DBS-170 | 0.1271 | 0.2343 | 0.23 | 0.52 |
| TS15-DBS-171 | 0.134 | 0.1923 | 0.21 | 0.76 |
| TS15-DBS-172 | 0.1225 | 0.166 | 0.24 | 0.72 |
| TS15-DBS-173 | 0.1515 | 0.1458 | 0.22 | 0.84 |
| TS15-DBS-174 | 0.138 | 0.1285 | 0.24 | 0.57 |
| TS15-DBS-175 | 0.1506 | 0.134 | 0.22 | 0.43 |
| TS15-DBS-176 | 0.127 | 0.1403 | 0.24 | 0.55 |
| TS15-DBS-177 | 0.1395 | 0.2676 | 0.24 | 0.78 |
| TS15-DBS-178 | 0.1408 | 0.1935 | 0.23 | 0.51 |
| TS15-DBS-179 | 0.1522 | 0.1705 | 0.22 | 0.55 |
| TS15-DBS-180 | 0.1247 | 0.1427 | 0.24 | 0.36 |
| TS15-DBS-181 | 0.158 | 0.126 | 0.24 | 0.11 |
| TS15-DBS-182 | 0.1376 | 4 | 0.24 | 2.42 |
| TS15-DBS-183 | 0.154 | 2.4234 | 0.23 | 0.64 |
| TS15-DBS-184 | 0.1451 | 0.1212 | 0.22 | 0.19 |
| TS15-DBS-185 | 2.9517 | 0.1124 | 17.38 | 0.41 |
| TS15-DBS-186 | 0.1914 | 3.2139 | 0.25 | 1 |
| TS15-DBS-187 | 0.1392 | 0.1151 | 0.23 | 0.19 |
| TS15-DBS-188 | 0.1419 | 0.1165 | 0.24 | 0.33 |
| TS15-DBS-189 | 0.1206 | 0.096 | 0.25 | 0.05 |
| TS15-DBS-190 | 0.1407 | 0.1498 | 0.21 | 0.14 |
| TS15-DBS-191 | 0.1393 | 0.1938 | 0.21 | 0.12 |
| TS15-DBS-192 | 0.1368 | 3.6195 | 0.28 | 6.29 |
| TS15-DBS-193 | 0.1225 | 0.1281 | 0.22 | 0.13 |
| TS15-DBS-194 | 0.1685 | 0.1289 | 0.2 | 0.2 |
| TS15-DBS-195 | 0.1576 | 0.0949 | 0.22 | 0.12 |
| TS15-DBS-196 | 3.119 | 0.1018 | 16.63 | 0.35 |
| TS15-DBS-197 | 2.961 | 0.1021 | 18.02 | 0.34 |
| TS15-DBS-198 | 0.1343 | 0.129 | 0.2 | 0.1 |
| TS15-DBS-199 | 0.1456 | 0.149 | 0.22 | 0.16 |
| TS15-DBS-200 | 0.1395 | 0.2302 | 0.23 | 0.68 |
| TS15-DBS-201 | 0.1272 | 0.1032 | 0.2 | 0.11 |
| TS15-DBS-202 | 0.1105 | 0.091 | 0.25 | 0.15 |
| TS15-DBS-203 | 0.1308 | 0.1521 | 0.2 | 1.02 |
| TS15-DBS-204 | 0.1035 | 0.1311 | 0.2 | 0.33 |
| TS15-DBS-205 | 0.1451 | 0.1266 | 0.19 | 0.34 |
| TS15-DBS-206 | 0.114 | 0.2168 | 0.24 | 0.85 |
| TS15-DBS-207 | 0.1178 | 0.2644 | 0.23 | 1.26 |
| TS15-DBS-208 | 0.111 | 0.1506 | 0.23 | 0.35 |
| TS15-DBS-209 | 0.1288 | 0.1919 | 0.24 | 0.63 |
| TS15-DBS-210 | 0.097 | 0.0981 | 0.24 | 0.28 |
| TS15-DBS-211 | 0.1601 | 0.1494 | 0.2 | 0.67 |
| TS15-DBS-212 | 0.143 | 0.1516 | 0.22 | 0.19 |
| TS15-DBS-213 | 0.237 | 0.1838 | 0.24 | 0.15 |
| TS15-DBS-214 | 0.1245 | 0.2346 | 0.23 | 0.11 |
| TS15-DBS-215 | 0.1104 | 0.1382 | 0.24 | 0.07 |
| TS15-DBS-216 | 0.1205 | 0.1353 | 0.25 | 0.18 |
| TS15-DBS-217 | 0.1213 | 0.1187 | 0.25 | 0.13 |
| TS15-DBS-218 | 0.0964 | 0.1256 | 0.23 | 0.25 |
| TS15-DBS-219 | 0.1329 | 0.117 | 0.25 | 0.28 |
| TS15-DBS-220 | 0.1246 | 0.1218 | 0.2 | 0.13 |
| TS15-DBS-221 | 0.1174 | 0.1347 | 0.23 | 0.23 |
| TS15-DBS-222 | 0.1166 | 0.2077 | 0.22 | 0.13 |
| TS15-DBS-223 | 0.1035 | 0.1603 | 0.22 | 0.12 |
| TS15-DBS-224 | 0.114 | 0.1346 | 0.22 | 0.17 |
| TS15-DBS-225 | 0.1087 | 0.1065 | 0.2 | 0.03 |
| TS15-DBS-226 | 0.0984 | 0.1147 | 0.23 | 0.17 |
| TS15-DBS-227 | 2.7804 | 0.1271 | 14.53 | 0.38 |
| TS15-DBS-228 | 0.1181 | 0.1238 | 0.22 | 0.19 |
| TS15-DBS-229 | 0.1252 | 4.0 | 0.23 | 11.52 |
| TS15-DBS-230 | 0.1557 | 4.0 | 0.25 | 19.04 |
| TS15-DBS-231 | 2.7504 | 0.146 | 14.06 | 0.35 |
| TS15-DBS-232 | 0.1303 | 0.1625 | 0.18 | 0.28 |
| TS15-DBS-233 | 0.1056 | 0.1326 | 0.18 | 0.34 |
| TS15-DBS-234 | 0.1023 | 0.1345 | 0.2 | 0.41 |
| TS15-DBS-235 | 0.1438 | 0.1182 | 0.19 | 0.49 |
| TS15-DBS-236 | 0.1163 | 0.1407 | 0.2 | 0.42 |
| TS15-DBS-237 | 0.164 | 0.2318 | 0.2 | 0.57 |
| TS15-DBS-238 | 0.1367 | 0.1545 | 0.2 | 0.51 |
| TS15-DBS-239 | 0.1166 | 0.1456 | 0.2 | 0.7 |
| TS15-DBS-240 | 0.1372 | 0.2607 | 0.19 | 0.77 |
| TS15-DBS-241 | 0.1411 | 0.1389 | 0.22 | 0.37 |
| TS15-DBS-242 | 0.1376 | 0.2005 | 0.25 | 0.6 |
| TS15-DBS-243 | 0.1064 | 0.1737 | 0.22 | 0.43 |
| TS15-DBS-244 | 0.1036 | 0.1188 | 0.23 | 0.12 |
| TS15-DBS-245 | 0.1137 | 0.1202 | 0.22 | 0.31 |
| TS15-DBS-246 | 0.1013 | 0.1347 | 0.24 | 0.24 |
| TS15-DBS-247 | 0.1355 | 0.2745 | 0.22 | 0.78 |
| TS15-DBS-248 | 0.1115 | 0.1652 | 0.2 | 0.29 |
| TS15-DBS-249 | 0.1533 | 0.1723 | 0.2 | 0.32 |
| TS15-DBS-250 | 0.1343 | 0.1561 | 0.21 | 0.47 |
| TS15-DBS-251 | 0.1228 | 0.147 | 0.2 | 0.39 |
| TS15-DBS-252 | 0.1098 | 0.131 | 0.23 | 0.37 |
| TS15-DBS-253 | 0.1052 | 0.1466 | 0.2 | 0.4 |
| TS15-DBS-254 | 0.1015 | 0.1324 | 0.23 | 0.29 |
| TS15-DBS-255 | 0.1054 | 0.1379 | 0.2 | 0.53 |
| TS15-DBS-256 | 0.1053 | 0.1297 | 0.22 | 0.32 |
| TS15-DBS-257 | 0.1693 | 0.1452 | 0.19 | 0.59 |
| TS15-DBS-258 | 0.1333 | 0.1361 | 0.18 | 0.49 |
| TS15-DBS-259 | 0.1247 | 0.1252 | 0.23 | 0.41 |
| TS15-DBS-260 | 0.1115 | 0.1297 | 0.2 | 0.24 |
| TS15-DBS-261 | 0.1214 | 0.1266 | 0.22 | 0.32 |
| TS15-DBS-262 | 0.1012 | 0.2035 | 0.22 | 0.58 |
| TS15-DBS-263 | 0.1088 | 0.144 | 0.21 | 0.46 |
| TS15-DBS-264 | 0.1234 | 0.1787 | 0.19 | 0.47 |
| TS15-DBS-265 | 0.1125 | 0.0863 | 0.21 | 0.04 |
| TS15-DBS-266 | 0.1131 | 0.1026 | 0.21 | 0.14 |
| TS15-DBS-267 | 0.0888 | 0.0786 | 0.23 | 0.04 |
| TS15-DBS-268 | 0.1001 | 0.1 | 0.23 | 0.09 |
| TS15-DBS-269 | 0.1069 | 0.0947 | 0.23 | 0.12 |
| TS15-DBS-270 | 0.0919 | 0.1004 | 0.23 | 0.07 |
| TS15-DBS-271 | 0.113 | 0.1448 | 0.21 | 0.18 |
| TS15-DBS-272 | 0.1081 | 0.1239 | 0.23 | 0.07 |
| TS15-DBS-273 | 0.1654 | 0.1938 | 0.23 | 0.29 |
| TS15-DBS-274 | 0.1094 | 0.188 | 0.24 | 0.34 |
| TS15-DBS-275 | 0.1382 | 0.189 | 0.22 | 0.23 |
| TS15-DBS-276 | 0.1179 | 0.1198 | 0.24 | 0.07 |
| TS15-DBS-277 | 0.1111 | 0.1351 | 0.22 | 0.09 |
| TS15-DBS-278 | 0.1065 | 0.1132 | 0.22 | 0.19 |
| TS15-DBS-279 | 0.1128 | 0.2481 | 0.22 | 0.34 |
| TS15-DBS-280 | 0.1509 | 0.1596 | 0.25 | 0.26 |
| TS15-DBS-281 | 0.1662 | 0.2142 | 0.23 | 0.46 |
| TS15-DBS-282 | 0.1254 | 0.1284 | 0.25 | 0.3 |
| TS15-DBS-283 | 0.1443 | 4.0 | 0.24 | 11.44 |
| TS15-DBS-284 | 0.1283 | 0.2393 | 0.23 | 0.31 |
| TS15-DBS-285 | 0.1041 | 0.137 | 0.21 | 0.18 |
| TS15-DBS-286 | 0.1807 | 0.134 | 0.22 | 0.24 |
| TS15-DBS-287 | 0.1629 | 0.1493 | 0.2 | 0.31 |
| TS15-DBS-288 | 0.1058 | 0.2275 | 0.24 | 0.09 |
| TS15-DBS-289 | 0.1338 | 0.1905 | 0.24 | 0.11 |
| TS15-DBS-290 | 0.1528 | 0.1708 | 0.25 | 0.15 |
| TS15-DBS-291 | 0.1217 | 4.0 | 0.24 | 12.77 |
| TS15-DBS-292 | 0.1272 | 0.1473 | 0.25 | 0.18 |
| TS15-DBS-293 | 0.1268 | 0.1822 | 0.24 | 0.31 |
| TS15-DBS-294 | 0.107 | 0.1262 | 0.24 | 0.17 |
| TS15-DBS-295 | 0.1131 | 0.1409 | 0.27 | 0.14 |
| TS15-DBS-296 | 0.1165 | 0.253 | 0.21 | 0.24 |
| TS15-DBS-297 | 0.1212 | 0.7834 | 0.22 | 0.31 |
| TS15-DBS-298 | 0.1273 | 0.1893 | 0.22 | 0.14 |
| TS15-DBS-299 | 0.1394 | 4.0 | 0.22 | 18.21 |
| TS15-DBS-300 | 0.0991 | 0.1322 | 0.24 | 0.14 |
| TS15-DBS-301 | 0.1111 | 0.1745 | 0.22 | 0.22 |
| TS15-DBS-302 | 0.1797 | 0.1493 | 0.21 | 0.13 |
| TS15-DBS-303 | 0.1324 | 0.1695 | 0.23 | 0.14 |
| TS15-DBS-304 | 0.1002 | 0.1692 | 0.24 | 0.14 |
| TS15-DBS-305 | 0.1273 | 0.1318 | 0.22 | 0.05 |
| TS15-DBS-306 | 0.1406 | 0.221 | 0.27 | 0.21 |
| TS15-DBS-307 | 0.1244 | 0.1001 | 0.23 | 0.11 |
| TS15-DBS-308 | 0.1082 | 0.122 | 0.22 | 0.19 |
| TS15-DBS-309 | 0.116 | 0.1353 | 0.21 | 0.26 |
| TS15-DBS-310 | 0.1888 | 0.1662 | 0.23 | 0.36 |
| TS15-DBS-311 | 0.1467 | 4.0 | 0.2 | 16.72 |
| TS15-DBS-312 | 0.1634 | 0.2351 | 0.25 | 0.17 |
| TS15-DBS-313 | 0.1007 | 0.1176 | 0.25 | 0.04 |
| TS15-DBS-314 | 0.1325 | 4.0 | 0.22 | 13.05 |
| TS15-DBS-315 | 0.1508 | 4.0 | 0.22 | 25.95 |
| TS15-DBS-316 | 0.1109 | 0.0899 | 0.24 | 0.01 |
| TS15-DBS-317 | 0.1191 | 0.1141 | 0.26 | 0.11 |
| TS15-DBS-318 | 0.1465 | 0.1205 | 0.23 | 0.26 |
| TS15-DBS-319 | 0.1321 | 0.1663 | 0.2 | 0.26 |
| TS15-DBS-320 | 0.1704 | 0.297 | 0.22 | 0.24 |
| TS15-DBS-321 | 0.1832 | 0.1702 | 0.23 | 0.29 |
| TS15-DBS-322 | 0.1216 | 0.1625 | 0.22 | 0.17 |
| TS15-DBS-323 | 0.1228 | 4.0 | 0.22 | 1.52 |
| TS15-DBS-324 | 0.179 | 0.1469 | 0.23 | 0.21 |
| TS15-DBS-325 | 0.1606 | 0.2211 | 0.22 | 0.29 |
| TS15-DBS-326 | 0.1242 | 0.2288 | 0.23 | 0.39 |
| TS15-DBS-327 | 0.1346 | 0.168 | 0.22 | 0.21 |
| TS15-DBS-328 | 0.1246 | 0.1439 | 0.22 | 0.28 |
| TS15-DBS-329 | 0.1282 | 0.1316 | 0.24 | 0.22 |
| TS15-DBS-330 | 0.1228 | 0.1377 | 0.23 | 0.29 |
| TS15-DBS-331 | 0.1198 | 0.1535 | 0.25 | 0.23 |
| TS15-DBS-332 | 0.1197 | 0.171 | 0.25 | 0.2 |
| TS15-DBS-333 | 0.1284 | 0.1847 | 0.24 | 0.19 |
| TS15-DBS-334 | 0.1102 | 0.1671 | 0.22 | 0.14 |
| TS15-DBS-335 | 0.1061 | 0.1156 | 0.23 | 0.11 |
| TS15-DBS-336 | 0.1194 | 0.1572 | 0.23 | 0.17 |
| TS15-DBS-337 | 0.1265 | 0.1913 | 0.24 | 0.14 |
| TS15-DBS-338 | 0.1565 | 0.1973 | 0.23 | 0.92 |
| TS15-DBS-339 | 0.11 | 0.1508 | 0.23 | 0.55 |
| TS15-DBS-340 | 0.1169 | 0.1965 | 0.2 | 0.82 |
| TS15-DBS-341 | 0.1238 | 0.1864 | 0.2 | 0.73 |
| TS15-DBS-342 | 0.114 | 0.2119 | 0.23 | 0.95 |
| TS15-DBS-343 | 0.1076 | 0.3266 | 0.24 | 0.25 |
| TS15-DBS-344 | 0.1268 | 4.0 | 0.22 | 17.14 |
| TS15-DBS-345 | 0.1157 | 0.129 | 0.22 | 0.09 |
| TS15-DBS-346 | 0.119 | 0.1542 | 0.22 | 0.17 |
| TS15-DBS-347 | 0.1061 | 4.0 | 0.24 | 7.75 |
| TS15-DBS-348 | 0.1217 | 4.0 | 0.23 | 12.41 |
| TS15-DBS-349 | 0.1258 | 4.0 | 0.22 | 21.67 |
| TS15-DBS-350 | 0.1218 | 0.1231 | 0.22 | 0.34 |
| TS15-DBS-351 | 0.1277 | 0.0677 | 0.22 | 0.28 |
| TS15-DBS-352 | 0.1269 | 0.2915 | 0.24 | 0.37 |
| TS15-DBS-353 | 0.1968 | 0.1743 | 0.28 | 0.17 |
| TS15-DBS-354 | 0.1381 | 0.184 | 0.2 | 0.31 |
| TS15-DBS-355 | 0.1175 | 0.1168 | 0.23 | 0.09 |
| TS15-DBS-356 | 0.1062 | 0.1028 | 0.22 | 0.1 |
| TS15-DBS-357 | 0.1601 | 0.1452 | 0.23 | 0.19 |
| TS15-DBS-358 | 0.1204 | 0.1562 | 0.24 | 0.21 |
| TS15-DBS-359 | 0.1228 | 0.1648 | 0.23 | 0.14 |
| TS15-DBS-360 | 0.1115 | 0.2422 | 0.22 | 0.21 |
| TS15-DBS-361 | 0.265 | 4.0 | 0.23 | 25.4 |
| TS15-DBS-362 | 0.1597 | 0.187 | 0.22 | 0.43 |
| TS15-DBS-363 | 0.1337 | 0.1057 | 0.25 | 0.1 |
| TS15-DBS-364 | 0.1353 | 0.1943 | 0.2 | 0.24 |
| TS15-DBS-365 | 0.1089 | 0.1169 | 0.2 | 0.17 |
| TS15-DBS-366 | 0.1252 | 0.1112 | 0.2 | 0.3 |
| TS15-DBS-367 | 0.1161 | 4.0 | 0.25 | 4.3 |
| TS15-DBS-368 | 0.1322 | 0.2491 | 0.23 | 0.24 |
| TS15-DBS-369 | 0.2063 | 0.2173 | 0.2 | 0.51 |
| TS15-DBS-370 | 0.1453 | 0.2016 | 0.23 | 0.17 |
| TS15-DBS-371 | 0.1349 | 0.1782 | 0.24 | 0.17 |
| TS15-DBS-372 | 0.1521 | 0.1776 | 0.2 | 0.24 |
| TS15-DBS-373 | 0.1822 | 0.1693 | 0.22 | 0.21 |
| TS15-DBS-374 | 0.1375 | 0.196 | 0.21 | 0.27 |
| TS15-DBS-375 | 0.1437 | 0.1781 | 0.22 | 0.14 |
| TS15-DBS-376 | 0.1188 | 0.2799 | 0.25 | 0.29 |
| TS15-DBS-377 | 0.1324 | 0.1966 | 0.22 | 0.19 |
| TS15-DBS-378 | 0.1291 | 0.1664 | 0.2 | 0.29 |
| TS15-DBS-379 | 0.1312 | 0.2773 | 0.23 | 0.34 |
| TS15-DBS-380 | 0.1173 | 0.152 | 0.24 | 0.15 |
| TS15-DBS-381 | 0.1398 | 0.1108 | 0.23 | 0.34 |
| TS15-DBS-382 | 0.1127 | 0.1934 | 0.22 | 0.32 |
| TS15-DBS-383 | 0.1223 | 0.2284 | 0.21 | 0.41 |
| TS15-DBS-384 | 2.6665 | 4.0 | 40.09 | 7.24 |
| TS15-DBS-385 | 0.1531 | 0.1531 | 0.22 | 0.56 |
| TS15-DBS-386 | 0.1087 | 0.1329 | 0.22 | 0.39 |
| TS15-DBS-387 | 0.1284 | 0.1233 | 0.22 | 0.29 |
| TS15-DBS-388 | 0.1026 | 0.1203 | 0.25 | 0.13 |
| TS15-DBS-389 | 0.0952 | 0.0744 | 0.22 | 0.09 |
| TS15-DBS-390 | 0.0975 | 0.1859 | 0.24 | 0.29 |
| TS15-DBS-391 | 0.1379 | 4.0 | 0.22 | 7.67 |
| TS15-DBS-392 | 0.1273 | 0.3111 | 0.22 | 0.24 |
| TS15-DBS-393 | 0.1675 | 4.0 | 0.2 | 12.7 |
| TS15-DBS-394 | 0.1172 | 0.1665 | 0.24 | 0.32 |
| TS15-DBS-395 | 0.125 | 0.1206 | 0.21 | 0.2 |
| TS15-DBS-396 | 0.1479 | 0.16 | 0.25 | 0.34 |
| TS15-DBS-397 | 0.118 | 0.1827 | 0.25 | 0.27 |
| TS15-DBS-398 | 0.1001 | 0.1308 | 0.22 | 0.16 |
| TS15-DBS-399 | 0.115 | 0.1516 | 0.21 | 0.13 |
| TS15-DBS-400 | 0.1064 | 0.2018 | 0.2 | 0.14 |
| TS15-DBS-401 | 0.1169 | 0.1472 | 0.23 | 0.2 |
| TS15-DBS-402 | 0.1159 | 0.2568 | 0.18 | 0.28 |
| TS15-DBS-403 | 0.1356 | 0.1264 | 0.24 | 0.16 |
| TS15-DBS-404 | 0.1166 | 0.1994 | 0.21 | 0.19 |
| TS15-DBS-405 | 0.1182 | 0.1722 | 0.25 | 0.17 |
| TS15-DBS-406 | 0.1031 | 0.1525 | 0.24 | 0.17 |
| TS15-DBS-407 | 0.1281 | 0.1984 | 0.19 | 0.31 |
| TS15-DBS-408 | 0.1053 | 0.1664 | 0.22 | 0.24 |
| TS15-DBS-409 | 0.108 | 0.1203 | 0.21 | 0.14 |
| TS15-DBS-410 | 0.1767 | 0.1761 | 0.19 | 0.29 |
| TS15-DBS-411 | 0.1362 | 4.0 | 0.22 | 11.18 |
| TS15-DBS-412 | 1.895 | 4.0 | 18.33 | 6.78 |
| TS15-DBS-413 | 0.1406 | 4.0 | 0.25 | 13.52 |
| TS15-DBS-414 | 0.1053 | 0.1338 | 0.24 | 0.24 |
| TS15-DBS-415 | 0.147 | 4.0 | 0.2 | 22.01 |
| TS15-DBS-416 | 0.1016 | 0.1353 | 0.23 | 0.17 |
| TS15-DBS-417 | 0.1003 | 0.158 | 0.24 | 0.07 |
| TS15-DBS-418 | 0.121 | 0.0982 | 0.21 | 0.17 |
| TS15-DBS-419 | 0.1147 | 0.1329 | 0.23 | 0.24 |
| TS15-DBS-420 | 0.1149 | 0.1604 | 0.21 | 0.24 |
| TS15-DBS-421 | 0.1344 | 0.1329 | 0.2 | 0.26 |
| TS15-DBS-422 | 0.1178 | 0.1861 | 0.19 | 0.27 |
| TS15-DBS-423 | 0.122 | 0.2027 | 0.2 | 0.16 |
| TS15-DBS-424 | 0.1145 | 0.2981 | 0.2 | 0.17 |
| TS15-DBS-425 | 0.1079 | 4.0 | 0.21 | 10.27 |
| TS15-DBS-426 | 0.1276 | 0.1809 | 0.23 | 0.16 |
| TS15-DBS-427 | 0.13 | 0.1451 | 0.25 | 0.11 |
| TS15-DBS-428 | 0.1208 | 0.1542 | 0.23 | 0.13 |
| TS15-DBS-429 | 0.12 | 0.1316 | 0.27 | 0.11 |
| TS15-DBS-430 | 0.0995 | 0.117 | 0.24 | 0.12 |
| TS15-DBS-431 | 0.1372 | 0.1661 | 0.25 | 0.12 |
| TS15-DBS-432 | 0.1074 | 0.2108 | 0.26 | 0.16 |
| TS15-DBS-433 | 0.1258 | 0.2493 | 0.23 | 0.19 |
| TS15-DBS-434 | 0.11 | 0.1511 | 0.46 | 0.08 |
| TS15-DBS-435 | 0.1144 | 0.1382 | 0.28 | 0.1 |
| TS15-DBS-436 | 0.1029 | 0.1431 | 2.16 | 0.04 |
| TS15-DBS-437 | 0.1048 | 0.1332 | 3.53 | 0.12 |
| TS15-DBS-438 | 0.1098 | 0.1651 | 0.22 | 0.29 |
| TS15-DBS-439 | 0.1124 | 0.3228 | 0.23 | 0.41 |
| TS15-DBS-440 | 0.1002 | 0.2547 | 0.2 | 0.31 |
| TS15-DBS-441 | 0.1028 | 0.1605 | 0.21 | 0.23 |
| TS15-DBS-442 | 0.0991 | 0.2036 | 0.22 | 0.29 |
| TS15-DBS-443 | 0.1117 | 0.1161 | 0.21 | 0.11 |
| TS15-DBS-444 | 0.1324 | 0.1369 | 0.2 | 0.22 |
| TS15-DBS-445 | 0.117 | 0.1617 | 0.21 | 0.22 |
| TS15-DBS-446 | 0.1002 | 0.1393 | 0.23 | 0.14 |
| TS15-DBS-447 | 0.1136 | 0.1982 | 0.22 | 0.27 |
| TS15-DBS-448 | 0.1168 | 4.0 | 0.24 | 4.5 |
| TS15-DBS-449 | 0.131 | 4.0 | 0.22 | 3.86 |
| TS15-DBS-450 | 0.115 | 4.0 | 0.22 | 4.09 |
| TS15-DBS-451 | 0.1051 | 3.7963 | 0.24 | 1.62 |
| TS15-DBS-452 | 0.145 | 3.2464 | 0.25 | 0.84 |
| TS15-DBS-453 | 0.1319 | 3.1752 | 0.25 | 0.91 |
| TS15-DBS-454 | 0.1324 | 0.6564 | 0.21 | 0.29 |
| TS15-DBS-455 | 0.1145 | 0.6667 | 0.23 | 0.41 |
| TS15-DBS-456 | 0.1245 | 4.0 | 0.23 | 14.94 |
| TS15-DBS-457 | 0.1427 | 4.0 | 4.31 | 14.71 |
| TS15-DBS-458 | 3.0755 | 0.3391 | 19.55 | 0.21 |
| TS15-DBS-459 | 0.096 | 4.0 | 2.44 | 3.26 |
| TS15-DBS-460 | 0.115 | 4.0 | 0.29 | 4.03 |
| TS15-DBS-461 | 0.1162 | 4.0 | 0.22 | 10.94 |
| TS15-DBS-462 | 0.1594 | 4.0 | 0.54 | 22.19 |
| TS15-DBS-463 | 0.1167 | 4.0 | 0.23 | 8.17 |
| TS15-DBS-464 | 2.8141 | 0.6467 | 37.94 | 0.99 |
| TS15-DBS-465 | 0.1548 | 4.0 | 0.27 | 7.41 |
| TS15-DBS-466 | 0.1814 | 4.0 | 1.91 | 10.89 |
| TS15-DBS-467 | 2.6384 | 0.3201 | 33.11 | 0.4 |
| TS15-DBS-468 | 2.4562 | 0.1227 | 9.16 | 0.14 |
| TS15-DBS-469 | 1.7424 | 0.134 | 1.5 | 0.11 |
| TS15-DBS-470 | 0.1211 | 4.0 | 0.36 | 2.01 |
| TS15-DBS-471 | 0.1614 | 4.0 | 1.24 | 2.04 |
| TS15-DBS-472 | 0.1041 | 3.9775 | 7.14 | 2.32 |
| TS15-DBS-473 | 0.2101 | 4.0 | 12.08 | 15.96 |
| TS15-DBS-474 | 0.1551 | 4.0 | 0.8 | 14.34 |
| TS15-DBS-475 | 0.1697 | 0.8445 | 0.94 | 0.34 |
| TS15-DBS-476 | 0.2324 | 3.8482 | 4.9 | 0.91 |
| TS15-DBS-477 | 0.1502 | 4.0 | 1.92 | 3.52 |
| TS15-DBS-478 | 0.1669 | 3.9986 | 0.22 | 3.24 |
| TS15-DBS-479 | 1.6093 | 4.0 | 4.11 | 2.34 |
| TS15-DBS-480 | 0.1738 | 4.0 | 0.55 | 20.65 |
| TS15-DBS-481 | 0.1286 | 3.2703 | 0.21 | 1.31 |
| TS15-DBS-482 | 0.1613 | 4.0 | 0.21 | 11.35 |
| TS15-DBS-483 | 2.6338 | 4.0 | 41.25 | 5.78 |
| TS15-DBS-484 | 0.1446 | 4.0 | 0.21 | 5.96 |
| TS15-DBS-485 | 0.1708 | 2.7906 | 0.24 | 0.68 |
| TS15-DBS-486 | 0.1826 | 4.0 | 0.23 | 19.48 |
| TS15-DBS-487 | 2.5401 | 3.9953 | 18.94 | 6.21 |
| TS15-DBS-488 | 0.1556 | 4.0 | 0.23 | 9.31 |
| TS15-DBS-489 | 2.8756 | 4.0 | 25.91 | 25.82 |
| TS15-DBS-490 | 0.1414 | 2.4867 | 0.23 | 0.7 |
| TS15-DBS-491 | 0.103 | 4.0 | 0.25 | 2.23 |
| TS15-DBS-492 | 0.1791 | 4.0 | 0.23 | 0.82 |
| TS15-DBS-493 | 0.2445 | 4.0 | 0.25 | 17.13 |
| TS15-DBS-494 | 0.1465 | 4.0 | 0.31 | 10.03 |
| TS15-DBS-495 | 0.2152 | 4.0 | 0.27 | 17.19 |
| TS15-DBS-496 | 0.1462 | 4.0 | 0.25 | 11.36 |
| TS15-DBS-497 | 0.1791 | 4.0 | 0.22 | 8.34 |
| TS15-DBS-498 | 0.1269 | 4.0 | 0.23 | 9.01 |
| TS15-DBS-499 | 0.1683 | 4.0 | 0.22 | 21.63 |
| TS15-DBS-500 | 0.1399 | 4.0 | 0.22 | 6.85 |
| TS15-DBS-501 | 1.039 | 3.2109 | 0.3 | 0.53 |
| TS15-DBS-502 | 0.1175 | 4.0 | 0.24 | 7.15 |
| TS15-DBS-503 | 0.1641 | 4.0 | 0.26 | 9.84 |
| TS15-DBS-504 | 0.1844 | 4.0 | 0.25 | 20.96 |
| TS15-DBS-505 | 0.1461 | 4.0 | 0.23 | 10.45 |
| TS15-DBS-506 | 0.1561 | 2.5197 | 0.23 | 2.84 |
| TS15-DBS-507 | 2.6245 | 0.1691 | 45.94 | 0.09 |
| TS15-DBS-508 | 1.6517 | 4.0 | 11.54 | 3.7 |
| TS15-DBS-509 | 0.1686 | 4.0 | 0.21 | 19.36 |
| TS15-DBS-510 | 0.1491 | 4.0 | 0.23 | 11.23 |
| TS15-DBS-511 | 0.2093 | 4.0 | 0.26 | 17.83 |
| TS15-DBS-512 | 0.1569 | 3.9599 | 0.22 | 1.62 |
| TS15-DBS-513 | 0.1446 | 0.1352 | 0.22 | 0.28 |
| TS15-DBS-514 | 0.1442 | 0.139 | 0.22 | 0.21 |
| TS15-DBS-515 | 0.1182 | 0.1614 | 0.2 | 0.2 |
| TS15-DBS-516 | 0.1601 | 0.2143 | 0.24 | 0.35 |
| TS15-DBS-517 | 0.1344 | 0.1536 | 0.25 | 0.12 |
| TS15-DBS-518 | 0.16 | 0.1666 | 0.23 | 0.45 |
| TS15-DBS-519 | 0.1625 | 0.172 | 0.23 | 0.49 |
| TS15-DBS-520 | 0.142 | 0.2037 | 0.22 | 0.45 |
| TS15-DBS-521 | 0.1762 | 0.1724 | 0.22 | 0.5 |
| TS15-DBS-522 | 0.2051 | 0.1783 | 0.26 | 0.41 |
| TS15-DBS-523 | 0.1281 | 0.1542 | 0.2 | 0.3 |
| TS15-DBS-524 | 0.1378 | 0.1879 | 0.24 | 0.32 |
| TS15-DBS-525 | 0.1531 | 0.1311 | 0.23 | 0.25 |
| TS15-DBS-526 | 0.175 | 0.1802 | 0.22 | 0.88 |
| TS15-DBS-527 | 0.1547 | 0.1996 | 0.24 | 0.68 |
| TS15-DBS-528 | 0.1399 | 0.1764 | 0.2 | 0.47 |
| TS15-DBS-529 | 0.1774 | 0.1555 | 0.22 | 0.44 |
| TS15-DBS-530 | 0.1731 | 0.1583 | 0.24 | 0.35 |
| TS15-DBS-531 | 0.113 | 0.1695 | 0.21 | 0.26 |
| TS15-DBS-532 | 0.1383 | 0.2087 | 0.24 | 0.23 |
| TS15-DBS-533 | 0.1431 | 0.1531 | 0.2 | 0.43 |
| TS15-DBS-534 | 0.1583 | 0.1751 | 0.21 | 0.5 |
| TS15-DBS-535 | 0.137 | 0.1284 | 0.22 | 0.28 |
| TS15-DBS-536 | 0.1558 | 4.0 | 0.23 | 15.46 |
| TS15-DBS-537 | 0.2019 | 4.0 | 0.23 | 17.95 |
| TS15-DBS-538 | 0.3292 | 4.0 | 0.24 | 18.67 |
| TS15-DBS-539 | 0.1341 | 4.0 | 0.23 | 11.85 |
| TS15-DBS-540 | 0.1425 | 0.2382 | 0.23 | 0.59 |
| TS15-DBS-541 | 0.137 | 0.1221 | 0.21 | 0.23 |
| TS15-DBS-542 | 0.1388 | 0.097 | 0.22 | 0.39 |
| TS15-DBS-543 | 0.1413 | 0.1562 | 0.22 | 0.71 |
| TS15-DBS-544 | 0.1723 | 3.9847 | 0.22 | 2.44 |
| TS15-DBS-545 | 0.2153 | 0.1984 | 0.23 | 0.71 |
| TS15-DBS-546 | 0.2208 | 0.1868 | 0.23 | 0.43 |
| TS15-DBS-547 | 0.1449 | 4.0 | 0.21 | 25.92 |
| TS15-DBS-548 | 0.1519 | 0.2656 | 0.22 | 0.91 |
| TS15-DBS-549 | 0.2804 | 4.0 | 0.23 | 17.35 |
| TS15-DBS-550 | 0.2641 | 4.0 | 0.24 | 13.91 |
| TS15-DBS-551 | 0.1626 | 3.8883 | 0.25 | 1.65 |
| TS15-DBS-552 | 0.1936 | 4.0 | 0.25 | 23.12 |
| TS15-DBS-553 | 0.1511 | 0.1699 | 0.2 | 0.76 |
| TS15-DBS-554 | 0.2214 | 4.0 | 0.23 | 20.85 |
| TS15-DBS-555 | 0.1319 | 4.0 | 0.25 | 12.61 |
| TS15-DBS-556 | 0.187 | 0.2384 | 0.2 | 0.6 |
| TS15-DBS-557 | 0.1971 | 4.0 | 0.27 | 14.94 |
| TS15-DBS-558 | 0.172 | 0.2295 | 0.2 | 0.45 |
| TS15-DBS-559 | 0.1705 | 0.1679 | 0.22 | 0.62 |
| TS15-DBS-560 | 0.2403 | 4.0 | 0.22 | 14.77 |
| TS15-DBS-561 | 0.1501 | 2.4883 | 0.22 | 1.15 |
| TS15-DBS-562 | 0.2505 | 4.0 | 0.22 | 16.88 |
| TS15-DBS-563 | 0.1376 | 0.2665 | 0.22 | 0.24 |
| TS15-DBS-564 | 2.8027 | 4.0 | 12.2 | 19.42 |
| TS15-DBS-565 | 0.2089 | 0.1822 | 0.24 | 0.16 |
| TS15-DBS-566 | 0.1616 | 0.1458 | 0.2 | 0.4 |
| TS15-DBS-567 | 0.1621 | 0.1375 | 0.2 | 0.41 |
| TS15-DBS-568 | 0.1487 | 0.1363 | 0.2 | 0.29 |
| TS15-DBS-569 | 0.1603 | 0.1605 | 0.22 | 0.4 |
| TS15-DBS-570 | 0.189 | 0.148 | 0.22 | 0.3 |
| TS15-DBS-571 | 0.1847 | 0.1385 | 0.19 | 0.23 |
| TS15-DBS-572 | 0.1471 | 0.1802 | 0.23 | 0.32 |
| TS15-DBS-573 | 0.1918 | 0.2402 | 0.22 | 0.74 |
| TS15-DBS-574 | 0.1526 | 0.1591 | 0.22 | 0.7 |
| TS15-DBS-575 | 0.1371 | 0.1732 | 0.22 | 0.84 |
| TS15-DBS-576 | 0.1982 | 0.1346 | 0.23 | 0.51 |
| TS15-DBS-577 | 0.1638 | 0.1641 | 0.22 | 0.42 |
| TS15-DBS-578 | 0.175 | 0.16 | 0.22 | 0.6 |
| TS15-DBS-579 | 0.1628 | 0.1306 | 0.2 | 0.53 |
| TS15-DBS-580 | 0.1427 | 0.1658 | 0.22 | 0.63 |
| TS15-DBS-581 | 0.1993 | 0.1574 | 0.21 | 0.66 |
| TS15-DBS-582 | 0.119 | 0.138 | 0.22 | 0.6 |
| TS15-DBS-583 | 0.0826 | 4.0 | 0.23 | 11.48 |
| TS15-DBS-584 | 0.0823 | 4.0 | 0.23 | 4.58 |
| TS15-DBS-585 | 0.0955 | 3.4246 | 0.26 | 0.8 |
| TS15-DBS-586 | 0.0905 | 3.717 | 0.24 | 1.68 |
| TS15-DBS-587 | 0.7488 | 4.0 | 7.71 | 4.29 |
| TS15-DBS-588 | 0.1282 | 4.0 | 0.24 | 2.77 |
| TS15-DBS-589 | 0.1002 | 4.0 | 0.23 | 22.37 |
| TS15-DBS-590 | 0.0918 | 4.0 | 0.24 | 23.15 |
| TS15-DBS-591 | 0.1049 | 4.0 | 0.24 | 8.66 |
| TS15-DBS-592 | 0.0975 | 3.4693 | 0.27 | 0.73 |
| TS15-DBS-593 | 0.0863 | 4.0 | 0.25 | 3.55 |
| TS15-DBS-594 | 0.0917 | 3.3965 | 0.24 | 0.64 |
| TS15-DBS-595 | 0.0922 | 4.0 | 0.25 | 0.63 |
| TS15-DBS-596 | 0.0849 | 4.0 | 0.23 | 0.9 |
| TS15-DBS-597 | 1.855 | 0.2949 | 51.09 | 0.18 |
| TS15-DBS-598 | 0.1105 | 3.5781 | 1.33 | 2.7 |
| TS15-DBS-599 | 0.1084 | 2.0715 | 0.24 | 1 |
| TS15-DBS-600 | 0.1069 | 3.5433 | 0.23 | 1.3 |
| TS15-DBS-601 | 0.1633 | 4.0 | 0.28 | 12.73 |
| TS15-DBS-602 | 0.1417 | 4.0 | 0.24 | 16.12 |
| TS15-DBS-603 | 0.1055 | 4.0 | 1.02 | 2.73 |
| TS15-DBS-604 | 2.1371 | 4.0 | 39.66 | 4.68 |
| TS15-DBS-605 | 0.1221 | 0.2604 | 0.24 | 0.39 |
| TS15-DBS-606 | 0.1048 | 0.1706 | 0.22 | 0.18 |
| TS15-DBS-607 | 0.0952 | 0.4042 | 0.2 | 0.27 |
| TS15-DBS-608 | 0.104 | 0.2709 | 0.21 | 0.21 |
| TS15-DBS-609 | 0.1168 | 0.2241 | 0.19 | 0.24 |
| TS15-DBS-610 | 0.136 | 0.2066 | 0.2 | 0.13 |
| TS15-DBS-611 | 0.1363 | 3.5548 | 1.67 | 2.76 |
| TS15-DBS-612 | 0.1063 | 0.1753 | 0.2 | 0.23 |
| TS15-DBS-613 | 0.1728 | 3.8639 | 0.24 | 0.56 |
| TS15-DBS-614 | 0.0954 | 1.002 | 0.25 | 0.17 |
| TS15-DBS-615 | 0.1229 | 4.0 | 0.24 | 7.63 |
| TS15-DBS-616 | 0.1046 | 4.0 | 0.22 | 1.34 |
| TS15-DBS-617 | 0.1251 | 4.0 | 0.22 | 6.03 |
| TS15-DBS-618 | 0.1094 | 1.0914 | 0.22 | 0.71 |
| TS15-DBS-619 | 3.218 | 0.2086 | 10.38 | 0.38 |
| TS15-DBS-620 | 2.7218 | 0.2155 | 18.41 | 0.49 |
| TS15-DBS-621 | 2.5591 | 3.2902 | 11.01 | 0.18 |
| TS15-DBS-622 | 3.316 | 4.0 | 24.12 | 2.55 |
| TS15-DBS-623 | 0.1349 | 4.0 | 0.25 | 21.92 |
| TS15-DBS-624 | 0.1949 | 4.0 | 0.23 | 11.31 |
| TS15-DBS-625 | 0.1194 | 2.2063 | 0.23 | 0.83 |
| TS15-DBS-626 | 0.1536 | 0.2314 | 0.2 | 0.17 |
| TS15-DBS-627 | 0.1472 | 0.27 | 0.2 | 0.22 |
| TS15-DBS-628 | 0.1467 | 0.2659 | 0.23 | 0.14 |
| TS15-DBS-629 | 0.1373 | 0.2021 | 0.19 | 0.14 |
| TS15-DBS-630 | 0.1127 | 0.2528 | 0.23 | 0.18 |
| TS15-DBS-631 | 0.1715 | 4.0 | 1.93 | 2.17 |
| TS15-DBS-632 | 2.4108 | 4.0 | 20.99 | 2.9 |
| TS15-DBS-633 | 0.2002 | 0.2795 | 0.22 | 0 |
| TS15-DBS-634 | 0.1354 | 0.2573 | 0.19 | 0.05 |
| TS15-DBS-635 | 0.1277 | 0.2484 | 0.2 | 0.1 |
| TS15-DBS-636 | 0.1543 | 0.2876 | 0.21 | 0.21 |
| TS15-DBS-637 | 0.104 | 0.1878 | 0.22 | 0.04 |
| TS15-DBS-638 | 0.128 | 0.224 | 0.19 | 0.12 |
| TS15-DBS-639 | 2.3631 | 0.5781 | 48.01 | 0.3 |
| TS15-DBS-640 | 0.1644 | 4.0 | 0.29 | 5.69 |
| TS15-DBS-641 | 0.157 | 0.3033 | 0.2 | 0.08 |
| TS15-DBS-642 | 0.141 | 0.1557 | 0.23 | 0.13 |
| TS15-DBS-643 | 0.1348 | 0.1849 | 0.22 | 0.12 |
| TS15-DBS-644 | 0.1589 | 0.1921 | 0.23 | 0.08 |
| TS15-DBS-645 | 0.1222 | 0.194 | 0.21 | 0.09 |
| TS15-DBS-646 | 0.1441 | 4.0 | 0.21 | 26.11 |
| TS15-DBS-647 | 0.1555 | 4.0 | 0.23 | 9.59 |
| TS15-DBS-648 | 0.1208 | 4.0 | 0.24 | 3.97 |
| TS15-DBS-649 | 0.1285 | 4.0 | 0.24 | 3.15 |
| TS15-DBS-650 | 1.7817 | 4.0 | 10.56 | 3.76 |
| TS15-DBS-651 | 1.4961 | 4.0 | 10.41 | 1.95 |
| TS15-DBS-652 | 0.1163 | 3.9988 | 0.25 | 4.92 |
| TS15-DBS-653 | 2.68 | 1.14 | 30.7 | 0.93 |
| TS15-DBS-654 | 2.3765 | 3.9563 | 20.01 | 5.58 |
| TS15-DBS-655 | 1.7771 | 4.0 | 10.15 | 4.07 |
| TS15-DBS-656 | 0.106 | 4.0 | 0.25 | 1.16 |
| TS15-DBS-657 | 0.1269 | 4.0 | 0.29 | 1.77 |
| TS15-DBS-658 | 0.1142 | 4.0 | 0.23 | 10.63 |
| TS15-DBS-659 | 0.5241 | 4.0 | 0.2 | 1.41 |
| TS15-DBS-660 | 0.118 | 3.7933 | 0.23 | 3.19 |
| TS15-DBS-661 | 0.1271 | 4.0 | 0.87 | 4.4 |
| TS15-DBS-662 | 0.5547 | 0.1186 | 0.58 | 0.09 |
| TS15-DBS-663 | 0.6513 | 0.1183 | 0.25 | 0.09 |
| TS15-DBS-664 | 0.1202 | 4.0 | 0.25 | 8.95 |
| TS15-DBS-665 | 3.2059 | 0.5994 | 29.12 | 4.21 |
| TS15-DBS-666 | 3.2778 | 0.0606 | 17.93 | 0.33 |
| TS15-DBS-667 | 3.1644 | 0.1834 | 17.75 | 0.43 |
| TS15-DBS-668 | 0.1168 | 4.0 | 0.27 | 19.36 |
| TS15-DBS-669 | 0.1237 | 4.0 | 0.25 | 18.79 |
| TS15-DBS-670 | 0.1246 | 3.6391 | 0.23 | 0.81 |
| TS15-DBS-671 | 2.4952 | 4.0 | 52.16 | 3.21 |
| TS15-DBS-672 | 0.171 | 3.9937 | 0.48 | 1.34 |
| TS15-DBS-673 | 2.0445 | 4.0 | 7.76 | 1.84 |
| TS15-DBS-674 | 2.5315 | 2.7158 | 41.17 | 1.24 |
| TS15-DBS-675 | 0.0958 | 4.0 | 0.23 | 2 |
| TS15-DBS-676 | 0.1181 | 4.0 | 0.21 | 9.22 |
| TS15-DBS-677 | 0.1039 | 0.1192 | 0.23 | 0.11 |
| TS15-DBS-678 | 0.1248 | 0.1253 | 0.23 | 0.14 |
| TS15-DBS-679 | 0.1319 | 0.1746 | 0.25 | 0.08 |
| TS15-DBS-680 | 0.1234 | 4.0 | 0.21 | 5.59 |
| TS15-DBS-681 | 0.1173 | 0.1456 | 0.24 | 0.09 |
| TS15-DBS-682 | 0.1597 | 0.1583 | 0.21 | 0.18 |
| TS15-DBS-683 | 0.1309 | 3.7917 | 0.22 | 0.7 |
| TS15-DBS-684 | 0.1104 | 4.0 | 0.22 | 12.6 |
| TS15-DBS-685 | 2.0336 | 4.0 | 13.81 | 4.35 |
| TS15-DBS-686 | 0.1067 | 3.9706 | 0.24 | 1.6 |
| TS15-DBS-687 | 0.1286 | 4.0 | 0.23 | 3.77 |
| TS15-DBS-688 | 2.6646 | 4.0 | 27.22 | 4.85 |
| TS15-DBS-689 | 0.1006 | 4.0 | 0.28 | 4.59 |
| TS15-DBS-690 | 0.1234 | 3.5029 | 0.23 | 1.28 |
| TS15-DBS-691 | 0.1678 | 4.0 | 0.23 | 19.57 |
| TS15-DBS-692 | 0.1113 | 4.0 | 0.22 | 4.02 |
| TS15-DBS-693 | 0.1116 | 3.9955 | 0.22 | 5.33 |
| TS15-DBS-694 | 0.1055 | 4.0 | 0.23 | 7.04 |
| TS15-DBS-695 | 0.1132 | 3.4616 | 0.23 | 0.74 |
| TS15-DBS-696 | 0.1416 | 4.0 | 0.24 | 5.17 |
| TS15-DBS-697 | 2.5766 | 4.0 | 27.85 | 9.27 |
| TS15-DBS-698 | 0.1113 | 4.0 | 0.25 | 5.5 |
| TS15-DBS-699 | 0.1097 | 3.9373 | 0.24 | 2.05 |
| TS15-DBS-700 | 0.1255 | 4.0 | 0.23 | 8.23 |
| TS15-DBS-701 | 0.121 | 4.0 | 0.2 | 4.97 |
| TS15-DBS-702 | 0.166 | 4.0 | 0.21 | 8.14 |
| TS15-DBS-703 | 0.1348 | 4.0 | 0.22 | 7.41 |
| TS15-DBS-704 | 0.1363 | 4.0 | 0.2 | 2.68 |
| TS15-DBS-705 | 0.1147 | 3.761 | 0.31 | 0.76 |
| TS15-DBS-706 | 2.5982 | 4.0 | 14.18 | 10.37 |
| TS15-DBS-707 | 0.111 | 4.0 | 0.56 | 4.14 |
| TS15-DBS-708 | 2.7163 | 0.3978 | 22.96 | 0.6 |
| TS15-DBS-709 | 0.4238 | 0.186 | 8.09 | 0.24 |
| TS15-DBS-710 | 0.109 | 4.0 | 5.0 | 6.9 |
| TS15-DBS-711 | 0.0987 | 4.0 | 1.17 | 8.56 |
| TS15-DBS-712 | 2.6784 | 0.5209 | 23.53 | 1.12 |
| TS15-DBS-713 | 0.1149 | 4.0 | 2.6 | 12.68 |
| TS15-DBS-714 | 2.8234 | 4.0 | 30.45 | 20.42 |
| TS15-DBS-715 | 2.5098 | 0.3357 | 6.95 | 0.13 |
| TS15-DBS-716 | 0.1484 | 4.0 | 0.26 | 1.1 |
| TS15-DBS-717 | 0.1352 | 4.0 | 0.24 | 5.99 |
| TS15-DBS-718 | 0.162 | 4.0 | 0.2 | 6.08 |
| TS15-DBS-719 | 2.6597 | 0.131 | 9.17 | 0.12 |
| TS15-DBS-720 | 0.1829 | 4.0 | 0.22 | 2.77 |
| TS15-DBS-721 | 2.6451 | 0.1357 | 0.22 | 0.16 |
| TS15-DBS-722 | 2.7408 | 0.2317 | 30.2 | 0.33 |
| TS15-DBS-723 | 0.1384 | 4.0 | 0.22 | 9.37 |
| TS15-DBS-724 | 0.1537 | 4.0 | 0.23 | 21.26 |
| TS15-DBS-725 | 0.1133 | 4.0 | 0.23 | 16.8 |
